# Anti-amphiphysin antibody-associated paraneoplastic brainstem encephalitis with pruritus and dysphagia as the first symptoms: A case report

**DOI:** 10.1097/MD.0000000000035325

**Published:** 2023-09-29

**Authors:** Jin Wang, Xiaokun Mi, Feng Ban, Jingxia Zhao

**Affiliations:** a Department of Neurology, The Fourth Hospital of Hebei Medical University, Jian Kang Road No.12, Shijiazhuang, China.

**Keywords:** anti-amphiphysin antibody, brainstem encephalitis, dysphagia, paraneoplastic neurologic syndromes (PNS), pruritus

## Abstract

**Rationale:**

Anti-amphiphysin antibodies are uncommonly detected in paraneoplastic neurologic syndromes (PNS), especially in patients with small cell lung cancer. Here, we report the first case of anti-amphiphysin antibody-associated PNS with pruritus and dysphagia as the first complaints.

**Patient concerns:**

The patient was a 58-year-old man who sought medical advice with a chief complaint of dysphagia and the lung occupancy. We found that he had developed progressive pruritus several months ago.

**Diagnoses:**

In the outer basal segment of the right lung lower lobe, PET-CT revealed small occupancies with hypermetabolism. Later, the pathology showed small cell lung cancer. And anti-amphiphysin antibodies were detected in serum. Above all, the patient’s symptoms improved significantly after antitumor treatment. Even neither of the 2 cranial enhancement MRIs showed any meaningful imaging signs, the above evidence could confirm the diagnosis of PNS.

**Interventions:**

The chemotherapy regimen was etoposide 0.1g d1-3+cisplatin 40 mg d1-3 (q3w). Paroxetine 20 mg/day was given to relieve the itching.

**Outcomes:**

After the treatment, the Watian water swallowing test dropped from grade 5 to grade 1, the intense itching also became tolerable.

**Lessons:**

Clinicians should consider diagnoses other than anxiety states or esophageal cancer in a patient with pruritus and dysphagia, such as PNS.

## 1. Introduction

Paraneoplastic neurologic syndromes (PNS) are a group of clinical syndromes in which the nervous system is involved due to the distal effects of the tumor. The primary mechanism is now thought to be immune-mediated cross-reactivity between tumor antigens and normal neuronal tissues rather than direct invasion, compression, or metastasis of the tumor.^[1]^ The target antigens that are attacked during the immune response are divided into 2 main groups. One is nuclear or cytoplasmic proteins such as Hu, Yo, and Ma2. The other is intracellular synaptic proteins including amphiphysin and 65 kDa glutamic acid decarboxylase.^[2]^ Our case report first describes paraneoplastic brainstem encephalitis as a result of anti-amphiphysin antibodies with pruritus and dysphagia as its initial symptoms.

## 2. Case description

**Figure 1.**
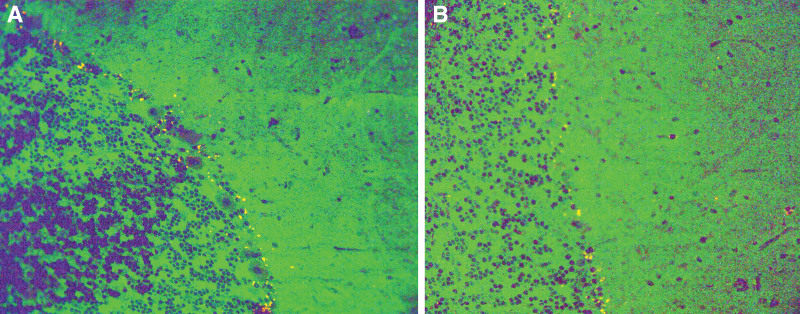
Detection of autoimmune microcephaly antibody.

A 58-year-old man visited the Fourth Hospital of Hebei Medical University (Hebei Provincial Cancer Hospital), in August 2022, with a 2-month history of dizziness and blurred vision. One month ago, he had dysphagia and scattered red herpes on the craniofacial region. At the local hospital, he was diagnosed with lung occupancy, while the pathology only indicated inflammatory and necrotic tissue. He was later seen at another hospital and underwent 2 cranial enhancement MRIs. Neither of them showed any meaningful imaging signs. In the outer basal segment of the right lung lower lobe, PET-CT revealed small occupancies with hypermetabolism. The neuron-specific enolase level was moderately elevated, and the paraneoplastic-related antibody profile in serum revealed anti-Amphiphysin antibodies IG+++(Fig. 1). Half a month ago, the dysphagia worsened leading to the placement of a gastric tube for enteral nutrition.

On admission, the Watian water swallowing test was grade 5. Other neurological facial examinations were normal. The blood routine and biochemical examinations did not reveal any abnormalities. No cancer cells were found in the cerebrospinal fluid. The routine and biochemical examinations of cerebrospinal fluid: protein ±; erythrocyte count:29 × 10^6^/L; leukocyte count: 3 × 10^6^/L. The chest puncture was performed on August 10, 2022, and the pathology showed small cell lung cancer (SCLC). In addition, he completed other examinations: Electromyography suggested peripheral nerve damage. Repetitive transcranial magnetic stimulation: low frequency, no decrement, high frequency, and no increment. The neostigmine test was negative. Eventually, the patient was thus clearly diagnosed with SCLC, paraneoplastic syndrome.

For the treatment of lung cancer, etoposide 0.1 g d1-3+cisplatin 40 mg d1-3 was given. After chemotherapy, the patient’s symptoms were dramatically relieved: on the first day, he could drink; on the second day, he could eat as usual, and the gastric tube was removed. Upon discharge, the Watian water swallowing test fell to grade 1.

Subsequently, the patient came to our hospital for chemotherapy every 21 days. After the third cycle, he suffered from severe pruritus around the left corner of the mouth and lower extremities (especially proximal and dorsal). It was insect-like, and the patient was unable to wear a mask, and his urination intervals were shortened to every half hour due to the irritation around his urethra. His quality of life was seriously affected by the intermittent pruritus, which was even worse at night. Upon reviewing the patient’s medical history, it was found that the pruritus had started as early as May. The patient’s first symptom was then clarified as pruritus of the face and lower extremities through the follow-up history.

Paroxetine 20 mg 1/day was given and relieved the pruritus. The effect could be observed in 7 days. Although no significant change in facial itching was seen, the pruritus of the lower extremities was significantly reduced.

## 3. Review and discussion

### 3.1. Anti-amphiphysin antibodies and paraneoplastic brainstem encephalitis

The fusion and reuptake of vesicles with the postsynaptic membrane during synaptic transmission is called synaptic vesicle endocytosis. Amphiphysin is involved in synaptic vesicle endocytosis by promoting the division of lattice-protein encapsulated vesicles, which leads to partial failure of message transmission when it is attacked by antibodies.^[3,4]^ They are a relatively uncommon autoantibody in patients with SCLC, regardless of the combination of PNS.^[5–7]^In 1993, De Camilli^[8]^ first reported 3 cases of female breast cancer patients with stiff-person syndrome. Their anti-amphiphysin antibodies are detected in sera. In addition to stiff-person syndrome, patients with anti-amphiphysin antibodies have been found to present with various neurological syndromes, such as encephalomyelitis, myoclonus, and cerebellar syndrome.^[5,9]^ Among these, brainstem encephalitis is an atypical manifestation of encephalomyelitis. As a result, patients often miss the best time for treatment. Ray ^[10]^described a case of anti-amphiphysin antibody-associated brainstem encephalitis with dizziness and hearing loss as the first symptoms. Her early treatment was delayed due to the visit to an otolaryngologist. To our knowledge, such patients with pruritus and dysphagia as the first symptoms have not been reported.

### 3.2. Pruritus and PNS

Three months prior to diagnosis, this patient had suffered from intractable pruritus, which was even misdiagnosed as an anxiety disorder. It suggests that chronic pruritus lasting more than 6 weeks may be the earliest manifestation of the paraneoplastic syndrome.^[11,12]^ Generally, paraneoplastic pruritus is caused by the lymphatic system, which occurs rarely in solid tumors.^[13]^ Insulinoma and colon cancer have both been reported to cause paraneoplastic pruritus,^[14,15]^ suggesting that paraneoplastic pruritus and neuroendocrine tumors are closely connected. Furthermore, SCLC with paraneoplastic pruritus symptoms associated with anti-Hu antibodies was reported,^[11]^ so as a patient with prostate cancer who ultimately diagnosed with paraneoplastic brainstem encephalitis.^[16]^ While anti-amphiphysin antibodies have rarely been linked to paraneoplastic pruritus in SCLC patients.

In layer I of the dorsal horn, the pruritic pathway forms a whole system mediated by inhibitory interneurons with the pain pathway. These interneurons may be the cellular basis for inhibiting pruritus by painful stimuli.^[17]^ Meanwhile, anti-amphiphysin antibodies are able to deactivate gabaergic interneurons by reducing the expression of the Na+/K+/2Cl2- cotransporter.^[18]^ Thus, it is speculated that it may be one of the mechanisms through which anti-amphiphysin antibodies cause paraneoplastic pruritus. Additionally, tumor tissue secretes excessive interleukin-31^[12]^ and 5-hydroxytryptamine.^[15]^ Paraneoplastic pruritus can also result from them.

Apparently, the most effective measure to control pruritus is to treat the underlying malignancy.^[12]^ In addition, molecularly speaking, a block can be made in the signaling pathway. Gabapentin is a structural analog of γ-aminobutyric acid and is thought to increase GABA concentration by affecting its metabolism^[19]^ and block the alpha2delta subunit of voltage-dependent calcium channels in dorsal horn postsynaptic cells.^[17]^ Antidepressive agents have also been shown to be applied to paraneoplastic pruritus by affecting serotonin and histamine levels.^[20]^ Despite not being widely used, they have shown significant efficacy.^[21]^

### 3.3. Dysphagia and PNS

The patient had progressive dysphagia, while 2 cranial enhancement MRIs showed no abnormality, and no esophageal neoplasm was seen on the endoscope. On the basis of clinical symptoms and physical examination, brainstem encephalitis was diagnosed. It is believed that antibodies may attack the patient’s swallowing reflex on the central pattern generator and the motor component of the swallowing reflex located in the brainstem. With antitumor treatment, the patient’s swallowing function improved significantly, confirming that the symptoms were an atypical manifestation of paraneoplastic syndrome.

## 4. Conclusion

It is recognized that symptoms such as pruritus and dysphagia do not entirely reflect anxiety states or esophageal cancer. Further screening for the possibility of paraneoplastic syndrome, especially SCLC and anti-amphiphysin antibodies, is absolutely needed.

## Acknowledgments

The authors wish to acknowledge Dr Ruixue Diao, Professor of Hebei Medical University, for the language instruction on our article.

## Author contribution

**Funding acquisition:** Jingxia Zhao.

**Writing – original draft:** Jin Wang.

**Writing – review & editing:** Xiaokun Mi, Feng Ban, Jingxia Zhao.
